# Melatonin reduces lung injury in type 1 diabetic mice by the modulation of autophagy

**DOI:** 10.1186/s12860-024-00505-9

**Published:** 2024-03-14

**Authors:** Jafar Rezaie, Mojtaba Jahanghiri, Reza Mosaddeghi- Heris, Sina Hassannezhad, Nima Abdyazdani, Afshin Rahbarghazi, Mahdi Ahmadi

**Affiliations:** 1 Solid Tumor Research Center, Cellular and Molecular Medicine Institute, Urmia University of Medical Sciences, Urmia, Iran; 2 Student Research Committee, Tabriz University of Medical Sciences, Tabriz, Iran; 3 Neurosciences Research Center, Tabriz University of Medical Sciences, Tabriz, Iran; 4 Stem Cell Research Center, Tabriz University of Medical Sciences, Tabriz, Iran; 5 Drug Applied Research Center, Tabriz University of Medical Sciences, Tabriz, Iran

**Keywords:** Autophagy, Diabetes, Lung injury, Melatonin

## Abstract

**Background:**

In recent years, the role of autophagy has been highlighted in the pathogenesis of diabetes and inflammatory lung diseases. In this study, using a diabetic model of mice, we investigated the expression of autophagy-related genes in the lung tissues following melatonin administration.

**Results:**

Data showed histopathological remodeling in lung tissues of the D group coincided with an elevated level of IL-6, Becline-1, LC3, and P62 compared to the control group (*p* < 0.05). After melatonin treatment, histopathological remodeling was improved D + Mel group. In addition, expression levels of IL-6, Becline-1, LC3, and P62 were decreased in D + Mel compared to D group (*P* < 0.05). Statistically significant differences were not obtained between Mel group and C group (*p* > 0.05).

**Conclusion:**

Our results showed that melatonin injection can be effective in the amelioration of lung injury in diabetic mice presumably by modulating autophagy-related genes.

## Background

**Fig. 1:**
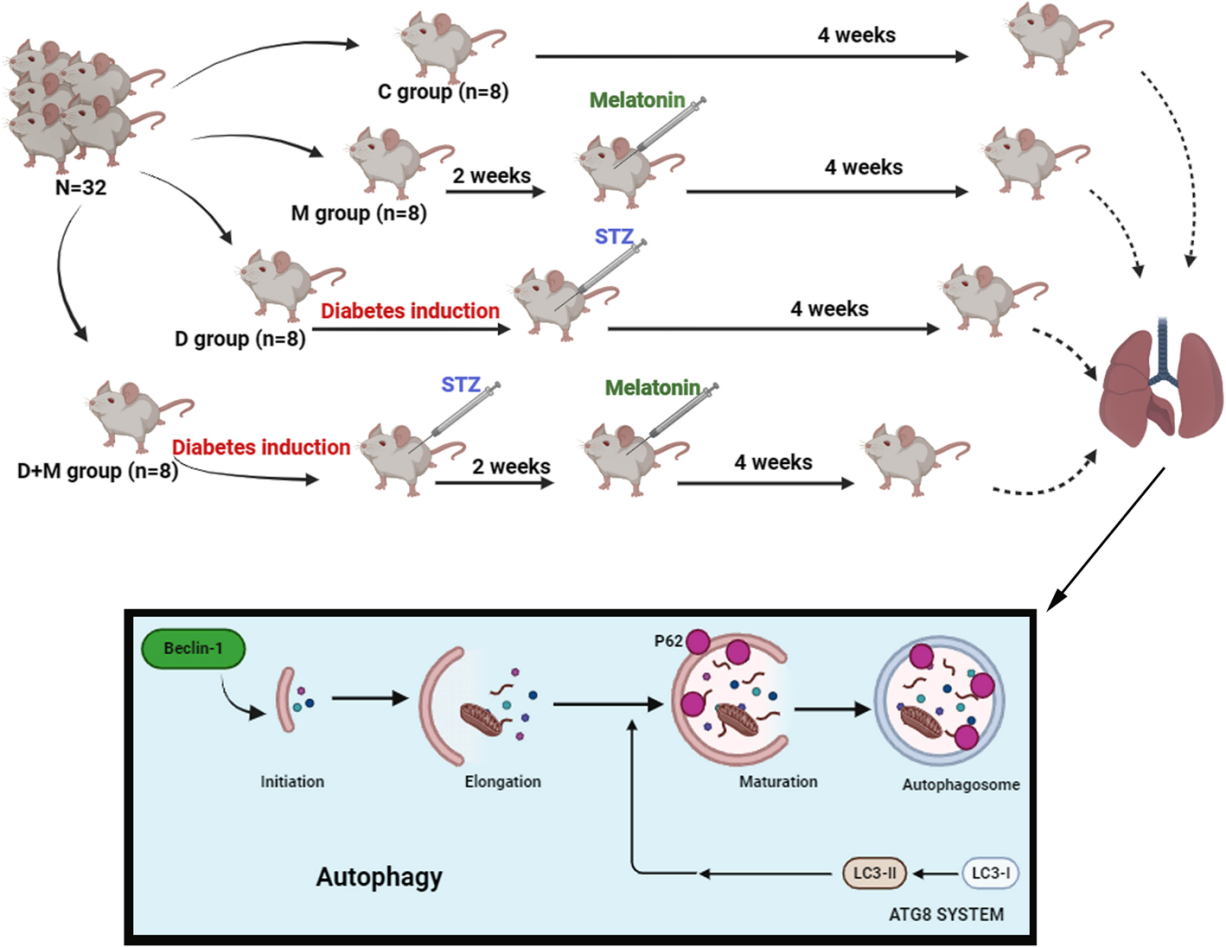
An overview of study design

Type 1 diabetes (T1D), characterized by destroyed insulin-producing cells, is a chronic immune-mediated condition with an autoimmune reason. It is believed that a genetically sensitive person is exposed to an assumed environmental component that results in a breakdown of immune control before developing cell autoantibodies. A decrease in insulin secretion, the emergence of hyperglycemia, and ultimately the development of T1D are caused by the destruction of cells [1]. The majority of diabetes studies have examined the effects of diabetes complications on other body tissues, including the heart, nervous system, and reproductive system [2, 3]. However, the precise mechanism of lung parenchymal damage in diabetes is still not fully understood. Undoubtedly, understanding the precise process of this damage can help manage and lessen the pulmonary difficulties that diabetic individuals experience [4, 5]. The lung parenchyma is vulnerable to injury in diabetics due to the high levels of elastin and collagen tissue, as well as the abundance of arterial beds in the lung tissue, on the one hand, and the presence of chronic oxidative stress and systemic inflammation in diabetes, on the other hand [6, 7]. Hyperglycemia's proinflammatory, proliferative, and oxidative characteristics have been demonstrated to play a significant part in how it affects the pulmonary vasculature, airways, and lung parenchyma [8]. A catabolic process known as "self-eating," autophagy includes the transfer of damaged organelles or misfolded proteins from the cytoplasm to the lysosome for destruction [9]. Basal autophagy normally plays a crucial role in maintaining cellular homeostasis; nevertheless, excessive autophagy results in a particular kind of cell death that plays a role in the etiology of numerous illnesses [10, 11]. Several factors such as Becline-1, LC3, and P62 contribute to initiating and progressing autophagy flux [12]. Melatonin, a crucial neuroendocrine hormone, is essential for reducing fibrosis, autophagy, mitochondrial fission, and insulin resistance in people with diabetes [13]. Today, melatonin's therapeutic and anti-inflammatory properties in chronic lung diseases have been proven [14–16]. There is evidence that melatonin can help avoid many diabetes consequences, such as damage to the liver, kidneys, and lungs, as well as cardiovascular disease [17]. This project was undertaken to design mice model of T1D and evaluate protective impact of melatonin on lung tissue histology and autophagy flux with focus on gene expression method (Fig. 1).

## Methods and Materials

### Animal issues

All experimental procedures were approved by Ethics Committee of Tabriz University of Medical Sciences according to National Institutes of Health for Laboratory Animal Care (revised 1996)with ethical number (No: IR.TBZMED.VCR.REC.1399.250).

### Experimental design

In this study we used thirty-two mice (initially weighing 25–30 g). Initially, mice kept foradaptation for 10 days, then were divided into four groups (*n* = 8) as follows: 1: Control group(C): received standard food and water. 2: Melatonin group (Mel): melatonin (Sigma-Aldrich, Steinheim, Germany)was injected intraperitoneally (3 mg/kg) for four weeks twice a week [1, 2]. 3: Diabetic group (D): received intraperitoneal injection of a single dose of streptozotocin (50 mg/kg). 4: Diabetic + Melatonin (D + Mel): two weeks after the induction of diabetes, melatonin was administered intraperitoneally (3 mg/kg) twice a week for four weeks(Fig. 1).

### Induction of T1D

After 8 h of fasting, T1D was induced by intraperitoneal injection of 50 mg/kg streptozotocin (STZ, Sigma-Aldrich, Steinheim, Germany) as a single dose (Fig. 1). In following, 72 h after the STZ injection, blood samples were obtained from the tail vein after overnight fasting and digital glucometer (Norditalia Elettromedicali S.r.I., San Martino della Battaglia, Italy) was used to measure glucose levels. Mice with serum glucose levels over 250 mg/dL were considered diabetic animals [3]. 48 h after the last injection, the animals were killed by injecting a high dose of ketamine and xylazine, and their lung tissue was coming out and analyzed for pathology (right lung) and gene expression (left lung).

### Histology of lung tissue

To examine the pathogenic impact of T1D on the pulmonary niche, formalin (10% w/v) was used to fix a sample of the right lung from each mouse. Next, samples were sliced into 5-μm thick pieces using a microtome and embedded in paraffin blocks. Hematoxylin and eosin (H&E) solution were used to stain the slides, and then monitored under a light microscope (Model: BX41; Olympus; Japan) as previously described [4]. Tissue damage including, bronchiolar epithelium degeneration and minor interstitial pneumonitis were evaluated.

### Real-time PCR

A quantitative real-time PCR assay was used to measure the mRNA levels of IL-6, LC3, Beclin-1, and p62. To conduct a gene expression analysis, a portion of a mouse's left lung was cut out and immediately frozen in liquid nitrogen for total RNA isolation according to the manufacturer's instructions of the RNA extraction kit (Yekta Tajhiz Azma Co, Iran). A Nanodrop spectrophotometer was used to measure RNA samples (NanoDrop-1000, USA). The cDNA Synthesis kit (Yekta Tajhiz Azma Co, Iran)was used to convert RNA into cDNA. Real time PCR (Corbett Life Science, Australia) and SYBR Green Master Mix (Yekta Tajhiz Azma Co, Iran)were used to perform reaction. Following extraction of ct values detected by PCR machine, the relative fold changeswere calculated using the 2^−ΔΔCT^ method after normalizing to the housekeeping gene, GAPDH. Table 1 lists the primers designed by Oligo 7 program (version 7.60).

**Table 1:** The list of primers used for real-time PCR analysis

Primer sequence (5'-3') Gene Forward Reverse		
LC-3	CATGCCGTCCGAGAAGACCT	GATGAGCCGGACATCTTCCACT
P62	GAGGCACCCCGAAACATGG	ACTTATAGCGAGTTCCCACCA
Beclin-1	TTGGCCAATAAGATGGGTCTGAA	TGTCAGGGACTCCAGATACGAGTG
IL-6	TACCACTTCACAAGTCGGAGGC	CTGCAAGTGCATCGTTGTTC
GAPDH	AACTTTGGCATTGTGGAAGG	ACACATTGGGGGTAGGAACA

### Data analysis

In the first step, the normality of the data was analyzed using Kolmogorov–Smirnov test.

Subsequently, data was analyzed using One-WayANOVA with Tukey–Kramer post hoc analysis and reported as means ± SEM. We considered a *p* value < 0.05 as statistically significant in GraphPad prism software (ver. 8).

## Results

### Melatonin diminished blood glucose levels in diabetic animals

**Fig. 2:**
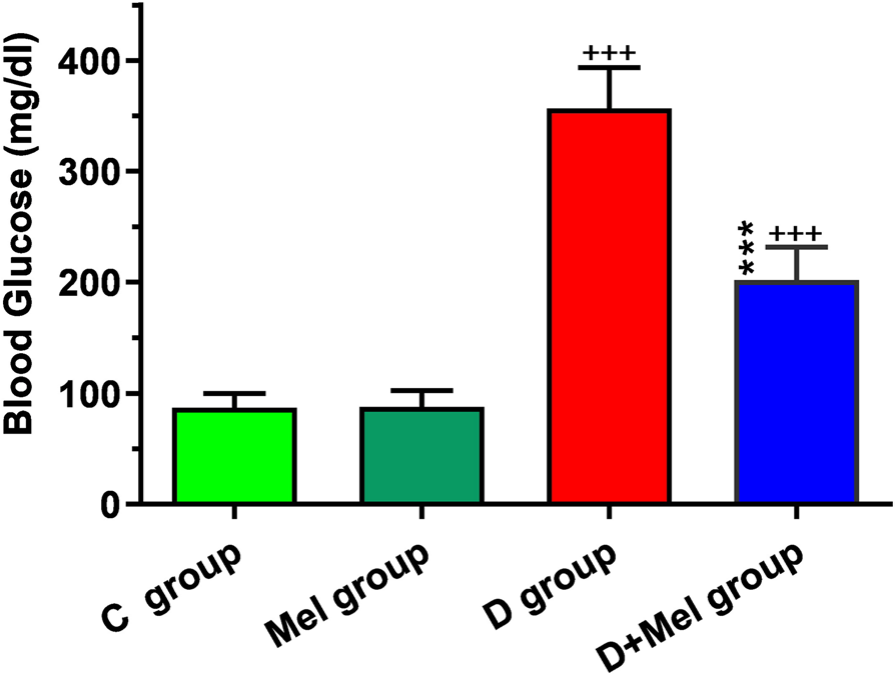
Measuring the blood glucose levels in control animals (C group), control animals received melatonin (Mel group), diabetic animals (D group), diabetic animals received melatonin (D + Mel group) (for each group, *n* = 8). Bars represent the mean ± SEM. Statistical differences between C and D groups: +  +  + ; *p* < 0. 001. Statistical differences between D group and D + Mel group:: +  +  + ; *p* < 0. 001

Based on our results, fasting blood glucose levels increased significantly 72 h after the STZ injection in diabetic groups compared to the C group (*p* < 0.001; Fig. 2). Melatonin led to a significant decrease in the glucose level in diabetic mice compared to the D group (*p* < 0.001; Fig. 2).

### Melatonin reduced pathological lesions in diabetic lungs

**Fig. 3:**
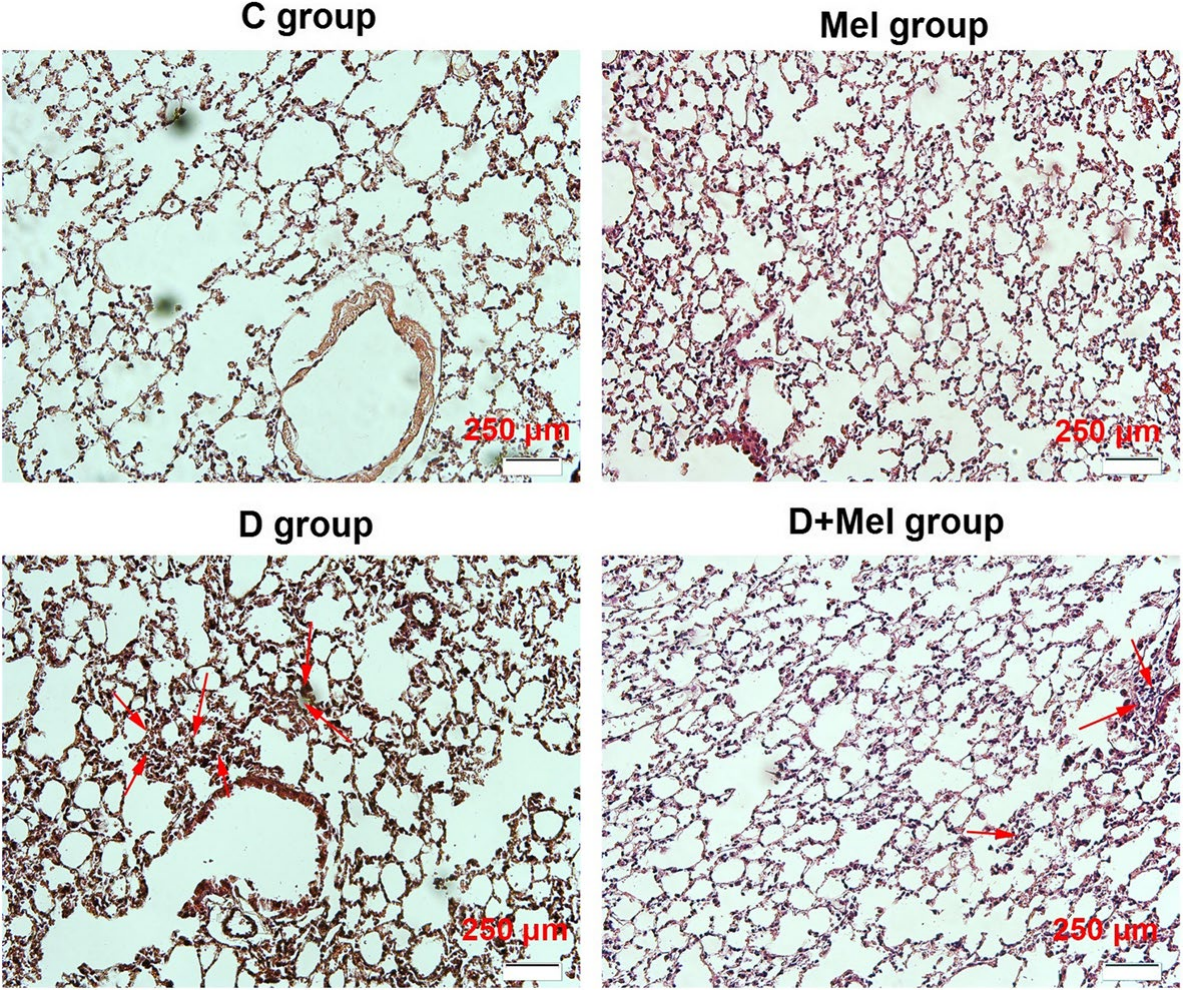
H & E staining of diabetic lung tissue. Imaging showed interstitial bronchopneumonia in diabetic lungs (red arrows). Melatonin reduced the damaging effects of diabetes on lung tissue

H & E staining revealed that induction of diabetes with STZ caused pathological changes in the pulmonary niche (Fig. 3). The images obtained from the light field microscope showed interstitial pneumonia (red arrows) in the diabetic lung tissue (groups D, D + Mel). As shown in Fig. 3, Melatonin reduced the severity of interstitial bronchopneumonia in D group lung tissue.

### Melatonin reduced the expression of the IL-6 gene within pulmonary tissue

**Fig. 4:**
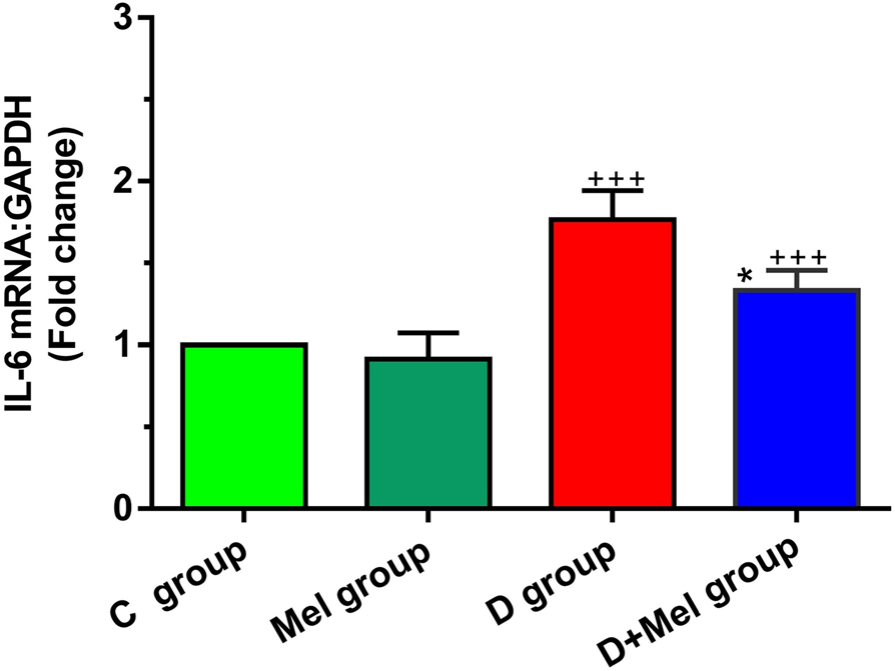
Measuring the transcription of IL-6 mRNA in the lung tissues of control animals (C group), control animals received melatonin (Mel group), diabetic animals (D group), diabetic animals received melatonin (D + Mel group) (for each group, *n* = 8). Bars represent the mean ± SEM. Statistical differences between C and D groups: +  +  + ; *p* < 0. 001. Statistical differences between D group and D + Mel group: *; *p* < 0.05

PCR assay showed that induction of diabetes with STZ caused an increase in the expression level of IL-6 in lung tissue compared to control mice (*p* < 0.001; Fig. 4). Compared to the D group, we found a statistically significant reduction in the expression of IL-6 in the D + Mel group (*p* < 0.05; Fig. 4). In addition, we did not find a significant difference between the M and C groups regarding the IL-6 gene (*p* > 0.05).

### Melatonin modulated autophagy status in DM1 mice

**Fig. 5:**
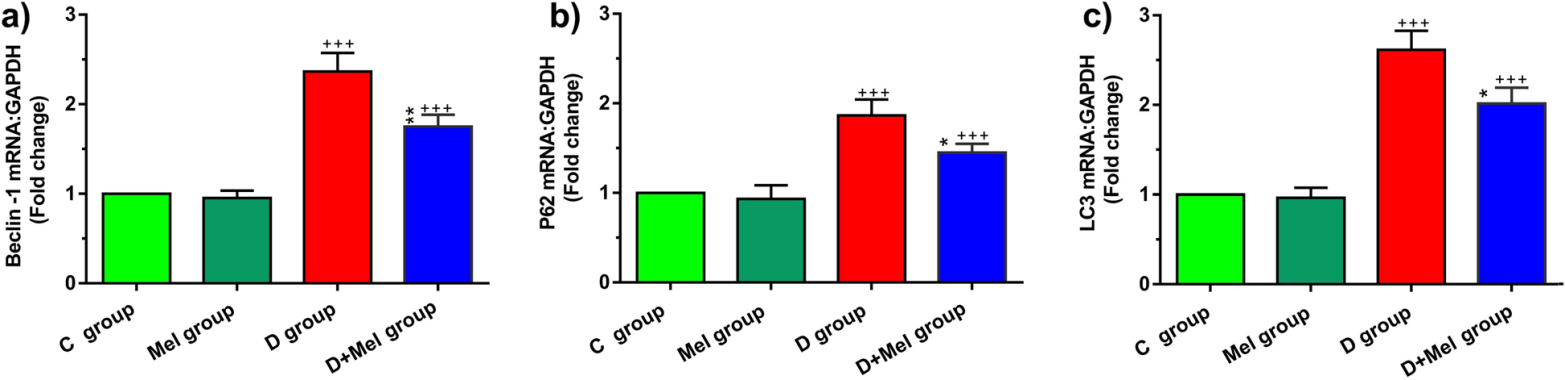
Measuring the transcription of Beclin-1 (a), P62 (b) and LC3 (c) mRNA in the lung tissues of different groups by real-time PCR. Bars represent the mean ± SEM. Statistical differences between C and D groups: +  +  + ; p < 0. 001. Statistical differences between D group and D + Mel group: *; p < 0.05 and **; p < 0.001

We also monitored the autophagy-related genes including Becline-1, LC3, and P62 in diabetic lungs (Fig. 5). Data indicated that the induction of diabetes could up-regulate Becline-1, LC3, and P62 genes against the C group (*p* < 0.001; Fig. 5a, b, and c). Compared to the D group, melatonin reduced the expression of these genes in the D + M group (*p* < 0.01 to *p* < 0.01; Fig. 5a, b, and c). The expression of these genes did not change in D + Mel compared to the C group (*p* > 0.05). These results showed that systemic injection of melatonin in the D + Mel group can bring, in part, the activity of the autophagy pathway closer to normal values.

## Discussion

Diabetes remains the most challenging health problem in the world [18]. Oxidative stress and inflammation play a central role in the diabetes. Therefore, this study set out with the aim of assessing the importance of melatonin in the treatment of complications associated with as melatonin showed antioxidants and anti-inflammatory properties [19, 20]. We measured the impact of melatonin on autophagy genes in the lung tissue of T1D mice.

As shown in Fig. 2, we observed interstitial pneumonia in lung samples of diabetic rats, which confirms histological changes in diabetic lung tissue. Furthermore, we found that the expression of IL-6 was up-regulated in lung tissues, indicating an inflammatory condition. Similarly, Aslani et al. showed that the expression of the IL-6 gene was elevated in the lung tissue of male rats suffering from allergic asthma [21]. Inflammation and pathological changes in the airways are the main characteristics of inflammatory lung diseases [22]. IL-6 is a proinflammatory cytokine that definitively promotes the development of insulin resistance and the pathogenesis of diabetes [23]**.** It seems that the vascular bed of the lung parenchyma and chronic inflammation in diabetes provide the key cause for the incidence of inflammation in the lungs and lung dysfunction [4, 7]. Therefore, these observations are a line with the previous studies confirming inflammation and pathological changes in lung tissues of diabetic animals [24, 25].

In keeping, after treatment with melatonin, we found a decrease in interstitial pneumonia and IL-6 expression in diabetic lung tissue. Melatonin is an antioxidant and anti-inflammatory agent that removes free radicals and ROS, preventing organelle damage within cells. There is evidence to suggest that melatonin participated in improving tissue damage and lessening inflammation by removing oxidative stress caused by diabetic conditions [26]. In our recent research, we found that intraperitoneal injection of melatonin for 4 weeks improved the heart function of Syrian mice model of diabetes [20]. Accordingly, confirmed that the serum level of melatonin hormone is low in people with diabetes and melatonin was very effective in improving the complications of diabetic patients, which may correlate with the anti-inflammatory and antioxidant activities of melatonin [13, 20].

In an experimental model of type 2 diabetes, it was found that administration of melatonin prevented the pro-inflammatory cytokines production including, IL-6, TNF-α, and CRP, inflammation as well as oxidative stress [27]. In STZ-induced diabetic rats, Farid et al. showed that melatonin administration reduced hyperlipidemia, hyperglycemia, and oxidative stress. Melatonin acted as an anti-inflammatory agent that inhibited oxidative stress and pro-inflammatory cytokines (IL-1β and IL-12) [28]. Generally, our results are well matched with the previous findings proposing that melatonin reduces tissue damage and inflammation in diabetic conditions. Furthermore, we evaluated the autophagy flux in lung tissues of different rats by analyzing autophagy-related genes and found that mRNA levels of LC3, Becline-1, and P62 in diabetic rats were higher than those of control and melatonin-treated rats. In other words, when diabetic rats received melatonin, the expression of these genes was inhibited, suggesting an inhibitory effect of Melatonin on autophagy flux. The activity of these genes and autophagy flux can be affected by several factors such as diet, high-fat foods, and metabolic stress such as oxidative stress [29, 30]. Autophagy plays a vital function in the complications of diabetes [31]. We know that, under diabetic conditions, metabolic inflammation is correlated with autophagy induced by oxidative stress [32], in addition, during inflammatory lung diseases, the level of autophagy is increased, thus lessening autophagy flux helps diseases improvement and reducing complications [33, 34]. More recently, Luo et al. reported that Melatonin improved renal injury in diabetic rats by regulating autophagy. They showed that Melatonin also reduced the protein expression levels of LC3II, P62 and COL-IV while the phosphorylation of AMPK was significantly augmented *both in vivo* and *in vitro* [35]. In addition, in type 2 diabetic rats, it was demonstrated that Melatonin lessened lung ischemia–reperfusion injury through SIRT3 signaling-dependent mitophagy [36]. In a study, it was demonstrated that diminished autophagy led to decreased cardiac injury in an animal model of type 1 diabetes [37]. It has previously been described that in STZ-induced type 1 diabetes or high glucose conditions, the autophagic activity of podocytes (kidney cells) is reduced, accompanied by a reduction in expression levels of autophagy-related proteins such as ATG12-5, Beclin-1, and LC3 [38, 39]. However, in a model of mice with diabetic cardiomyopathy, the administration of melatonin promoted autophagy flux within cardiac tissues, which improved cardiac remodeling and dysfunction [40].

Overall, we observed that melatonin reduced the expression of inflammation factor, IL-6 and improved pathological condition within lung tissues, which coincided with a reduced autophagic gene expression. Melatonin treatment resulted in a decrease in the expression level of autophagic genes in diabetic animals, whose levels were close to those of control. Therefore, in our opinion, increased autophagy during inflammation and tissue damage in diabetic animals was reduced after melatonin administration, confirming the antioxidant and anti-inflammatory impacts of melatonin [41, 42]. This study has gone some way towards enhancing our understanding of the function of melatonin in improving T1D lungs, and we hope that further works will prove our theory by measuring molecular signaling pathways behind crosstalk between inflammation responses and autophagy flux in T1D lungs. To be honest, the current study faces some limitations.. Here, we did not western blot analysis of autophagy-related markers. It is also recommended to evaluate the protein levels of factors related to apoptosis to better understand the mechanism of melatonin's effects in reducing pulmonary complications under diabetic conditions in future studies.

## Conclusion

The evidence from this study suggests that melatonin could ameliorate inflammation and pathological lesions in the lung tissues of T1D mice. At the same time, melatonin could reduce expression levels of autophagic genes and close them to those of control mice, indicating a correlation to diminished inflammation and pathological changes. Our preliminary data suggest that melatonin could be taken advantage of ameliorating T1D-associated complications, however, additional experimental investigations are desirable to confirm.

## Data Availability

The datasets are available from the corresponding author upon reasonable request.

## Abbreviations

T1D: Type 1 diabetes
STZ: Streptozotocin
